# Data on the relationship between lamotrigine and levetiracetam serum/plasma levels and toxicity: Experience at an academic medical center

**DOI:** 10.1016/j.dib.2021.107555

**Published:** 2021-11-08

**Authors:** Kelly E. Wood, Kendra L. Palmer, Matthew D. Krasowski

**Affiliations:** a Stead Family Department of Pediatrics, University of Iowa Stead Family Children's Hospital, Iowa City, IA 52242, USA; b Department of Pathology, University of Iowa Hospitals and Clinics, 200 Hawkins Drive, C-671 GH, Iowa City, IA 52242, USA

**Keywords:** Cardiotoxicity, Drug monitoring, Lamotrigine, Levetiracetam, Neurotoxicity syndromes, Pharmacokinetics

## Abstract

Lamotrigine and levetiracetam are second-generation anti-epileptic drugs used for the management of seizure disorders and some other medical conditions. In the related research article using retrospective data from an academic medical center, we analyzed 5046 samples originating from 1930 unique patients that had lamotrigine drug levels performed on serum/plasma and 4359 samples from 2451 patients that had levetiracetam drug levels performed. The data in this article provides the patient demographic, clinical location at time of drug level, and specific lamotrigine or levetiracetam serum/plasma drug level for all patients. For those instances with lamotrigine drug level greater than 14.0 mg/L or levetiracetam drug level of 80 mg/L or higher, additional data from chart review includes: indication for ordering the drug level, two main presenting signs or symptoms at time of drug level, timing of drug level (random, trough, peak, or unknown), changes in drug dosing following the drug level, concomitant therapy with valproic acid (lamotrigine only), and details related to drug overdose (if applicable). The analyzed data is provided in the supplementary tables included in this article. Volumes of test ordering by year is included in a figure. The dataset reported is related to the research article entitled “Correlation of Elevated Lamotrigine and Levetiracetam Serum/Plasma Levels with Toxicity: A Long-Term Retrospective Review at an Academic Medical Center” [K. E. Wood, K. L. Palmer, M.D. Krasowski, Correlation of elevated lamotrigine and levetiracetam serum/plasma levels with toxicity: A long-term retrospective review at an academic medical center, Toxicol. Rep. (2021) 8:1592-1598]

## Specifications Table

**Table:** 

Subject	Medicine and Dentistry
Specific subject area	Pathology and Medical Technology
Type of data	Supplementary tables Figure
How data were acquired	Retrospective chart and data review from laboratory analysis performed at an academic medical center
Data format	Raw and Analyzed
Parameters for data collection	Retrospective data was obtained from the electronic medical record (Epic, Inc.) covering the time period between August 1, 1996 through November 15, 2018 for lamotrigine and between January 1, 2005 and November 15, 2018 for levetiracetam. Detailed chart review was performed if lamotrigine drug level was greater than 14.0 mg/L or levetiracetam drug level was 80 mg/L or higher. The study had approval as a retrospective study from the University of Iowa Institutional Review Board.
Description of data collection	There were a total of 5046 samples originating from 1930 unique patients that had lamotrigine drug levels performed on serum/plasma and a total of 4359 samples from 2451 patients that had levetiracetam drug levels performed. Laboratory testing includes analysis of lamotrigine and levetiracetam drug levels on serum/plasma samples. Data includes patient demographic (age in years, sex), clinical location at time of drug level (outpatient, inpatient, emergency department), and specific lamotrigine or levetiracetam serum/plasma drug levels in mg/L for all patients. For those instances with lamotrigine drug level greater than 14.0 mg/L or levetiracetam drug level of 80 mg/L or higher, additional data from chart review includes: indication for ordering the drug level, two main presenting signs or symptoms at time of drug level, timing of drug level (random, trough, peak, or unknown), changes in drug dosing following the drug level, concomitant therapy with valproic acid (lamotrigine only), details related to drug overdose (if applicable), and details related to those patients who expired on same inpatient admission as an elevated lamotrigine or levetiracetam level.
Data source location	Iowa City, Iowa, United States of America
Data accessibility	One figure is included within the paper. 4 Supplementary files are deposited in Mendeley. Data identification number: 10.17632/fxbmcb8czp.1 Direct URL to data: https://data.mendeley.com//datasets/fxbmcb8czp/1
Related research article	Author's name: Kelly E. Wood, Kendra L. Palmer, Matthew D. Krasowski Title: Correlation of elevated lamotrigine and levetiracetam serum/plasma levels with toxicity: A long-term retrospective review at an academic medical center Journal: Toxicol Rep 8:1592-1598, 2021. DOI: https://doi.org/10.1016/j.toxrep.2021.08.005

## Value of the Data

• The data provided is of value with wider use of second-generation anti-epileptic drugs and more cases of potential toxicity.
• Clinicians, other researchers, or personnel in clinical laboratories might find this data useful as a reference for comparison.
• Our data set would serve as a starting point for researchers interested in future investigations of second-generation anti-epileptic drug toxicity.
• The data is of value as there is limited published data involving the relationship of lamotrigine and levetiracetam serum/plasma drug levels and clinical toxicity.
• The data provide information for a total of 9405 measurements in 4120 unique patients.

## 1 Data Description

**Fig. 1:**
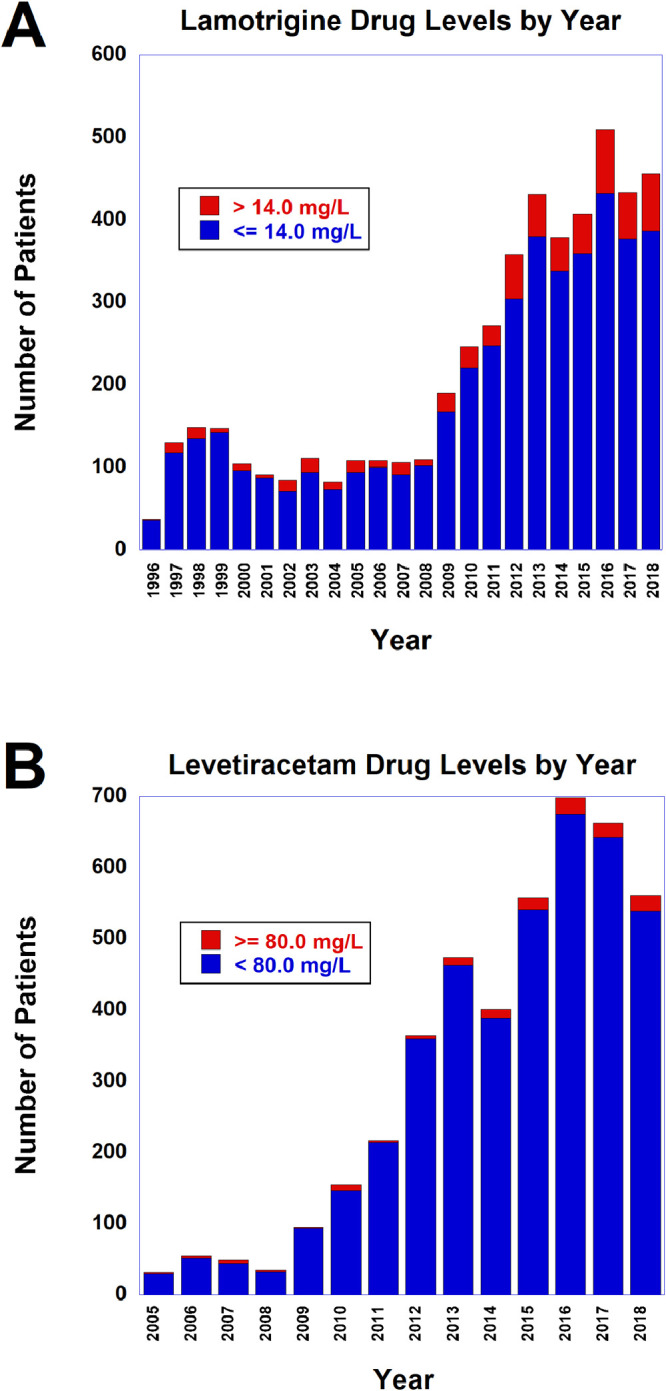
Number of drug levels for (A) lamotrigine and (B) levetiracetam by calendar year. For lamotrigine, the volumes are subdivided into > 14.0 mg/L (red) or ≤ 14.0 mg/L (blue). For levetiracetam, the volumes are subdivided into ≥ 80.0 mg/L (red) or < 80.0 mg/L (blue) (For interpretation of the references to color in this figure legend, the reader is referred to the web version of this article.).

In this retrospective study, we compiled detailed data on 9405 samples originating from 4120 unique patients that had lamotrigine or levetiracetam serum/plasma drug levels performed at an academic medical center central clinical laboratory. A total of 261 patients had both lamotrigine and levetiracetam drug levels performed in the retrospective timeframe. Therapeutic use of second-generation anti-epileptic drugs such as lamotrigine and levetiracetam has expanded markedly in the last two decades [1], [2], [3]. With increasing clinical use, more cases will be encountered with potentially toxic blood levels [4], [5], [6], [7], [8], [9], [10], [11]. Existing data on lamotrigine and levetiracetam toxicity has been mainly from case series and analysis of poison center data [5,[7], [8], [9], [10],[12], [13], [14], [15], [16]. The availability of enzyme immunoassays for measuring lamotrigine and levetiracetam serum/plasma levels allows for more clinical laboratories to do this testing onsite as opposed to referring to external reference laboratory [17,18]. The raw data are included in Supplementary file 1 (all lamotrigine drug levels), Supplementary file 2 (lamotrigine drug levels greater than 14.0 mg/L, with additional data from chart review), Supplementary file 3 (all levetiracetam drug levels), and supplementary file 4 (levetiracetam drug levels of 80 mg/L or higher, with additional data from chart review). Fig. 1 shows volumes of drug levels by calendar year, showing both all levels and the subset of levels defined as elevated (lamotrigine drug levels greater than 14.0 mg/L or levetiracetam drug levels of 80 mg/L or higher).

• Supplementary file 1: Data for 5046 measurements of lamotrigine serum/plasma drug levels on 1930 unique patients (1068 female, 862 male). Specific data fields include: location/unit at time of drug level testing (outpatient, inpatient, emergency department), sex (as officially recorded in electronic medical record), age in years, and lamotrigine drug level in mg/L.
• Supplementary file 2: Data for 597 measurements on 293 unique patients (163 female, 130 male) for which the lamotrigine serum/plasma drug level was greater than 14.0 mg/L. Specific data elements are the same as for Supplementary file 1 with additional data from chart review: indication for ordering the specific drug level (known overdose-intentional or accidental, possible symptoms of drug toxicity, routine check not related to pregnancy, routine check in pregnancy or post-partum, seizures, or unknown), two major presenting clinical signs and symptoms at time of drug level (agitation/aggression, altered mental status, asymptomatic, ataxia, cardiac symptoms, coma, dizziness, drooling, dry mouth, dysarthria, fatigue, headache, known ingestion leading to overdose, nausea/vomiting, night sweats, nystagmus, paranoia, paresthesias, psychosis, respiratory distress, seizures, tremors/twitches/jerks, unknown, vision changes, weakness, or weight loss), timing of drug level (random/unknown, peak, trough, other specific timing), changes in drug dosing following drug levels (decreased/temporarily held/discontinued maintenance dose, increased maintenance dose, maintained maintenance dose, or unknown), and concomitant valproic acid prescription at time of drug level (no or yes), and whether patient expired during same inpatient admission as the lamotrigine drug level.
• Supplementary file 3: Data for 4359 measurements of levetiracetam serum/plasma drug levels on 2451 unique patients (1225 female, 1226 male). Specific data fields include: location/unit at time of drug level testing (outpatient, inpatient, emergency department), sex (as officially recorded in electronic medical record), age in years, and levetiracetam drug level in mg/L.
• Supplementary file 4: Data for 134 measurements on 106 unique patients (33 female, 73 male) for which the levetiracetam serum/plasma drug level was 80 mg/L or higher. Specific data elements are the same as for Supplementary file 3 with additional data from chart review: indication for ordering the specific drug level (known overdose-intentional or accidental, possible symptoms of drug toxicity, routine check not related to pregnancy, routine check in pregnancy or post-partum, seizures, or unknown), two major presenting clinical signs and symptoms at time of drug level (agitation/aggression, altered mental status, asymptomatic, ataxia, cardiac symptoms, coma, dizziness, drooling, dry mouth, dysarthria, fatigue, headache, known ingestion leading to overdose, nausea/vomiting, night sweats, nystagmus, paranoia, paresthesias, psychosis, respiratory distress, seizures, tremors/twitches/jerks, unknown, vision changes, weakness, or weight loss), timing of drug level (random/unknown, peak, trough, other specific timing), changes in drug dosing following drug levels (decreased/temporarily held/discontinued maintenance dose, increased maintenance dose, maintained maintenance dose, or unknown), and whether patient expired during same inpatient admission as the levetiracetam drug level.

## 2 Experimental Design, Materials and Methods

Lamotrigine and levetiracetam serum/plasma levels were determined at a commercial reference laboratory (ARUP Laboratories, Salt Lake City, UT, USA) until July 8, 2011. Starting July 9, 2011, lamotrigine and levetiracetam were analyzed at the University of Iowa Hospitals and Clinics central clinical laboratory by enzyme immunoassay (ARK Diagnostics Lamotrigine Assay and Levetiracetam Reagent, respectively) on Roche Diagnostics c502 analyzers. Epic Reporting Workbench (RWB) [19], a reporting tool within the electronic medical record, was used to capture all cases where lamotrigine or levetiracetam drug levels had been performed within the retrospective timeframe. For the instances where the lamotrigine drug level was greater than 14.0 mg/L or the levetiracetam drug level was 80 mg/L or higher, the authors performed detailed chart review for the indication for ordering the specific drug level, clinical signs and symptoms at time of drug level, timing of drug level, changes in drug dosing following the drug level, concomitant valproic acid prescription at time of drug level (for lamotrigine cohort only), details on instances of intentional or accidental drug overdose, and details on patient deaths that occurred during inpatient admission as a high lamotrigine or levetiracetam drug level.

## Ethics Statement

The analyses had approval by the University of Iowa Institutional Review Board (protocol # 201812703) as a retrospective project. The research was carried out in accordance with The Code of Ethics of the World Medical Association (Declaration of Helsinki)

## CRediT authorship contribution statement

**Kelly E. Wood:** Formal analysis, Conceptualization, Writing – original draft, Writing – review & editing, Methodology. **Kendra L. Palmer:** Formal analysis, Writing – original draft, Writing – review & editing. **Matthew D. Krasowski:** Formal analysis, Conceptualization, Writing – original draft, Writing – review & editing, Methodology, Supervision.

## Declaration of Competing Interest

The authors declare that they have no known competing financial interests or personal relationships that could have appeared to influence the work reported in this paper.
